# Septins, a cytoskeletal protein family, with emerging role in striated muscle

**DOI:** 10.1007/s10974-020-09573-8

**Published:** 2020-01-18

**Authors:** Mónika Gönczi, Beatrix Dienes, Nóra Dobrosi, János Fodor, Norbert Balogh, Tamás Oláh, László Csernoch

**Affiliations:** 1 Department of Physiology, Faculty of Medicine, University of Debrecen, Debrecen, 4012 Hungary; 2 Doctoral School of Molecular Medicine, University of Debrecen, Debrecen, 4012 Hungary; 3 Center of Experimental Orthopaedics, Saarland University, 66421 Homburg, Saar Germany

**Keywords:** Septin, Oligomer assembly, Cellular function, Striated muscle

## Abstract

Appropriate organization of cytoskeletal components are required for normal distribution and intracellular localization of different ion channels and proteins involved in calcium homeostasis, signal transduction, and contractile function of striated muscle. Proteins of the contractile system are in direct or indirect connection with the extrasarcomeric cytoskeleton. A number of other molecules which have essential role in regulating stretch-, voltage-, and chemical signal transduction from the surface into the cytoplasm or other intracellular compartments are already well characterized. Sarcomere, the basic contractile unit, is comprised of a precisely organized system of thin (actin), and thick (myosin) filaments. Intermediate filaments connect the sarcomeres and other organelles (mitochondria and nucleus), and are responsible for the cellular integrity. Interacting proteins have a very diverse function in coupling of the intracellular assembly components and regulating the normal physiological function. Despite the more and more intense investigations of a new cytoskeletal protein family, the septins, only limited information is available regarding their expression and role in striated, especially in skeletal muscles. In this review we collected basic and specified knowledge regarding this protein group and emphasize the importance of this emerging field in skeletal muscle biology.

## Introduction: discovery and history of septins

The first discovery of septins was almost 50 years ago in the budding yeast *Saccharomyces cerevisiae* by Hartvell (1971) when they examined the genetic control and genes involved in cell division and cytokinesis. Four cell division cycle genes (cdc3, 10, 11, and 12) and a set of homologous proteins encoded by these genes were found to be associated with filaments at the cytoplasmic face of the plasma membrane in the mother-bud neck (Longtine et al. 1996). They also showed that temperature-sensitive mutation of these genes caused defective cytokinesis as it blocked specific steps in the cell division, namely that mutant strains developed multiple elongated buds, but did not separate from the parent cell. Electron microscopy analysis identified 10 nm thick filaments that encircled the septating bud neck (Byers and Goetsch 1976). Protein products of the regulatory genes were demonstrated as a fluorescently labelled ring within the septation area and were named septins by Kim et al. (1991).

**Fig. 1:**
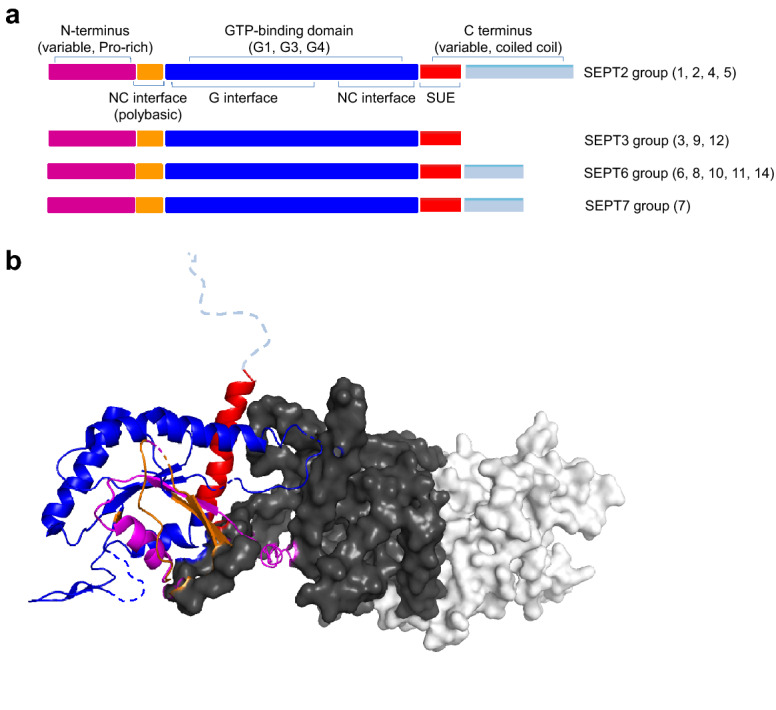
Shematic presentation of domains in the different septin subgroups (SUE: Septin Unique Elements) (**a**). Crystallographic data was used to generate a two-dimensional structural picture of SEPT7-SEPT6-SEPT2 trimers (PDB code:2QAG) using PyMol Software (**b**). SEPT2 is presented in white, SEPT6 in grey, while SEPT7 structure is shown more detailed; determined α-helices, β-sheets and linker regions within the different domains are presented, while dashed lines are used when proper information are missing from crystal structure

Since their discovery in yeast, homologous sequence proteins have been described in almost all types of eukaryotic cells. Septins are 30–65 kDa, evolutionarily highly conserved proteins that are considered as the fourth cytoskeletal component (Mostowy and Cossart 2012). All septins have multiple domains, and these domains possess a general organisation with the central Ras-like GTPase/GTP-binding domain (Vetter and Wittinghofer 2001), the N-terminal variant proline rich domain (PRD), the lipid binding polybasic region (PBR), the septin unique element (SUE) and the C-terminal coiled-coil (CC) region which has been shown to participate in protein–protein interactions (Casamayor and Snyder 2003; Versele and Thorner 2004). Septins belong to the family of P-loop GTPases (Weirich et al. 2008) with an alfa-beta core consisting of conserved G1 (or P-loop,“GxxxxGKS/T”), G3 (“DxxG”), and G4 (N/TKxD replaced by AKAD in septins) motifs for GTP binding and hydrolysis (Sirajuddin et al. 2009) (Fig. 1a). The encoded 13 septins in humans are classified into four homology groups SEPT2 (SEPT1, SEPT2, SEPT4, SEPT5), SEPT3 (SEPT3, SEPT9, SEPT12), SEPT6 (SEPT6, SEPT8, SEPT10, SEPT11, SEPT14), and SEPT7 (Hall et al. 2005). Septin oligomers have been recognized as a filamentous structure within the cytoplasm and show association with the cell membrane (Bertin et al. 2010; Zhang et al. 1999; Tanaka-Takiguchi et al. 2009), actin filaments (Kinoshita et al. 2002), and also with microtubules (Sellin et al. 2011; Surka et al. 2002; Nagata et al. 2003; Bowen et al. 2011; Kremer et al. 2005; Spilitois et al. 2008). These interactions influence septin assembly into filaments (Kinoshita et al. 2002; Bridges et al. 2014).

The expression of certain septin isoforms seems to be ubiquitous (SEPT2, SEPT7, and SEPT9) (Hall et al. 2005; Cao et al. 2007; Connolly et al. 2011), others (SEPT4, SEPT8, SEPT10, and SEPT11) are expressed extensively, however they are missing from some tissues, and further isoforms (SEPT1, SEPT3, SEPT12, and SEPT14) show tissue specific expression. As cytoskeletal components, septins have important roles in various cellular processes, including cell mobility (Kelley et al. 2015), apoptosis (Zhang et al. 2016; Fung et al. 2014), endocytosis (Hall and Russell 2012; Barve et al. 2018), and in determining cell shape (Mostowy et al. 2011; Kremer et al. 2007) within a wild range of organisms, such as yeast, drosophila, and mammals. Septin assemblies have been shown to act as scaffolds at the plasma membrane to regulate the distribution/recruitment of membrane-bound proteins promoting their functional interaction (Kinoshita 2006) and can also serve the compartmentalization of discrete cellular domains (Hall and Russell 2012). As a scaffold they can regulate the distribution of surface receptors (Kinoshita et al. 2004) and a clathrin-mediated endocytosis (Veiga and Cossart 2006; Mostowy et al. 2011, 2012). Septins are involved in establishing membrane rigidity and so regulating cell shape and directional cell movement (Gilden et al. 2012). Septin scaffold can also regulate vesicle fusion (Estey et al. 2010), microtubule-dependent transport in the cytoplasm (Bowen et al. 2011; Spilitois et al. 2008), has essential role during host–pathogen interactions and autophagy (Mostowy and Cossart 2009; Mostowy et al. 2011). They have a function in axon dynamics (Ageta-Ishihara et al. 2013), axon growth (Bai et al. 2013), chromosome segregation (Spilitois et al. 2005), cytokinesis (Menon et al. 2014), and dendrite formation (Xie et al. 2007), but also regulate cell motility (Tooley et al. 2009) and DNA repair (Connolly et al. 2011).

Little is known about their tissue specific functions, however, apart from the ubiquitous functions listed in the “Cellular function of septin proteins” section below, their contribution to the development, maintenance, and diseases of different organs are widely examined (Dolat et al. 2014b). The involvement of septin isoforms in neurological syndromes like Alzheimer’s and Parkinson’s disease (Kinoshita et al. 1998; Ihara et al. 2003), Down´s syndrome, schizophrenia, and different types of cancer such as leukemia (Elhasid et al. 2004), lymphoma, skin, breast (Zhang et al. 2016), colon, ovarian (Burrows et al. 2003; Scott et al. 2005) has already been proved. In this review we summarize information and data regarding the expression of septins in organisms from different level of phylogenetics, the knowledge about oligomerization of the septin isoforms and regulatory possibilities of their assembly, the morphological and functional interaction of septins and other cytoskeletal components and membranes, the cellular functions of septin organizations, and the involvement of septin assemblies in muscle type tissues. We collected thriving information of septins in several tissues that imply their contribution to proper muscle function.

## Phylogenetics, evolution

Septins are present in at least some representatives of every eukaryotic supergroup, with the possible exception of the Excavata. However, the structure, assembly, and biological roles of septins outside of the opisthokonts (animals, fungi, and their close relatives) largely remain unknown (Onishi and Pringle 2016). In non-opisthokonts, Nishihama et al. (2011) revealed septin genes in *Chlamydomonas reinhardtii* and *Nannochloris bacillaris* and in two other chlorophyte algae, *Volvox carteri* (one gene) and *Chlorella variabilis* (two genes); in the brown alga *Ectocarpus siliculosus* (five genes); and in two ciliates, *Tetrahymena thermophila* and *Paramecium tetraurelia*. These groups represent lineages that diverged from one another, and from the opisthokonts, very early in eukaryotic evolution. No septins have been found in protozoa and plants so far (Onishi and Pringle 2016).

Originated from their common unicellular ancestor about 1.2 billion years ago, opisthokonts inherited a set of genes which, undergoing duplications, deletions and other modifications, resulted in a morphological and physiological diversity (Ruiz-Trillo et al. 2007). In accordance with this, the number of septins and the coding genes vary between organisms (Nishihama et al. 2011; Pan et al. 2007). Based on phylogenetic investigations, all septins from fungi, microsporidia, and animals were clustered into five groups: Group 1 and Group 2 contain septin sequences from fungi and animals, Group 3 and Group 4 contain septin sequences from fungi and microsporidia, and Group 5 contains septin sequences from filamentous fungi (Pan et al. 2007). Further evolutionary analysis separated all metazoan (multicellular eukaryotic organisms) septin proteins into four subgroups: SEPT2, SEPT3, SEPT6, SEPT7 (Cao et al. 2007). Considering their important roles in morphogenesis, septin proteins might have contributed to the evolution of opisthokont complexity and diversity (Mostowy and Cossart 2012). Most opisthokont septins contain a strongly predicted coiled-coil domain at their C-termini (Longtine et al. 1996; Pan et al. 2007; Momany et al. 2008). This structure is absent in all of the non-opisthokont septins except for *C. variabilis*. Septin groups, including septins with coiled-coil domains, were ancient in the opisthokonts, suggesting that not only septin heterooligomers but also higher order filaments were part of the ancestral cellular tool kit of both animals and fungi. The presence of a hydrophobic (possibly transmembrane) domain, only in non-opisthokont septins lacking the coiled-coil domain, suggests that these motifs represent alternative evolutionary solutions in protein anchoring and/or protein–protein interaction (Nishihama et al. 2011).

In *Saccharomyces cerevisiae* septins have been identified as the major constituents of the bud-neck filaments, which have essential roles in cytokinesis (Nishihama et al. 2011). In this species 7 members of the septin family were identified, including cdc10, cdc3, cdc11, cdc12, shs1, which are expressed during vegetative growth, and spr3 and spr28, which are expressed in a temporally limited manner during spore formation and are involved in developing prospore wall. Interestingly, deletion of the spr3 or spr28 genes results in no obvious phenotype, and a double mutant has minimal defects in sporulation, suggesting that there is a compensation mechanism by the other septins (Kinoshita 2003a, b).

Septins were also identified in the phylogenetically distant (Kurtzman 1994) fission yeast *Schizosaccharomyces pombe*. The two yeast species are also morphogenetically different: instead of budding, *S. pombe* cells grow by extending the ends of the cylindrical cell and then divide by medial septum formation. This process partly depends on actin and associated proteins forming a contractile ring. Six septins (spn1p–spn6p) have been identified in *S. pombe*; spn1p appears to function in cell division, in a late stage of septum formation or in a localized cell-wall dissolution leading to the separation of the daughter cells (Longtine et al. 1996).

*Caenorhabditis elegans* has 2 septin members (unc-59, unc-61). The *C. elegans* mutants of unc-59 and unc-61 exhibit minimal defects in embryonic cytokinesis, but abnormalities in postembryonic morphogenesis occur in multiple organs, including germ-cell defects, egg-laying failure, and deformities in the male tail and sensory neurons. Further evidence suggests that *C. elegans* septins are required for the proper formation and structural integrity of the somatic gonad (Nguyen et al. 2000).

*Drosophila melanogester* has 5 septin members (pnut, sept1, sept2, sept4, sept5). Protein localization and phenotype of the pnut mutant *Drosophila* significantly contributed to our understanding of septin function. Pnut-null mutant larvae have severely reduced cell number, with multinucleated cells in the brain, and they die shortly after pupation. Mutant embryos lacking the pnut function from both the mother and the zygote, have abnormal organization of actin rings in the late cellularization stage of embryogenesis and extensive morphological defects during gastrulation (Kinoshita 2003a, b).

The presence of the representative homologue of the SEPT2, SEPT3, SEPT6, and SEPT7 subgroups in the non-vertebrate chordate *Ciona intestinalis* suggests that the emergence of the four septin subgroups described by Cao et al. (2007) occurred prior to the divergence of vertebrates from invertebrates, and the expansion of the number of septin genes in vertebrates was caused by mainly the duplication of pre-existing genes rather than by the appearance of a new septin subgroup (Cao et al. 2007). Humans have 13 septin members: 1–12 and 14 (Nishihama et al. 2011; Pan et al. 2007). The orthologues (homologous gene sequences) of most human septins existed in zebrafish, which suggests that the human septin gene tool kit was mainly formed before the fishes and land vertebrates became separated. The evolutionary rate within this septin family in mammalian lineage varies significantly, human SEPT1, SEPT10, SEPT12, and SEPT14 display a relatively elevated evolutionary rate compared with other septin members (Cao et al. 2007).

## Oligomerization of septin monomers

**Fig. 2:**
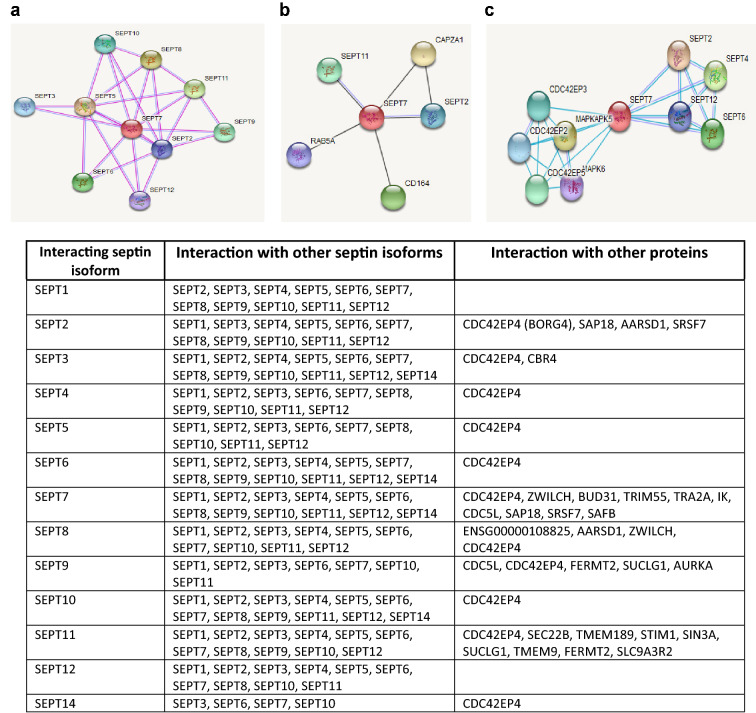
Interacting proteins with SEPT7 according the functional protein association network STRING. Settings of the interaction search was highest confidence (≥ 0.9) from active experimental (**a**), co-expression (**b**), or database (**c**) sources. Table shows interaction partners of all human septin isoforms identified experimentally either from the different septin subgroups or as other proteins. Information was collected from STRING using high confidence (≥ 0.7) setting

Septins form oligomeric complexes made up of different septin subgroup members, which are able to create higher-order structures, such as filaments, sheets, or rings involved in several biological processes (Weirich et al. 2008; Bertin et al. 2010). More and more experimental data are available regarding the interaction between septins and other proteins (Fig. 2).

The oligomeric complexes of paralogous septins contain four, six, or eight subunits, depending on the organism (Field et al. 1996; John et al. 2007; Sirajuddin et al. 2007; Bertin et al. 2008), forming heterooligomeric building blocks that assemble into filaments via longitudinal and lateral interactions (Weirich et al. 2008). These heterooligomeric complexes are formed by the interaction of G-domains using two interactive surfaces. One interface includes the N- and C-terminal extensions (NC-Interface) and the other one consists of the guanine nucleotide binding site and the β-hairpin insertion (G-interface). Within the heterooligomeric protein complexes G and NC interfaces alternate, the structure and order of the septin monomers determine the non-polar properties of the filaments. The stability of the interaction between two septin subunits depends on the presence of the guanine nucleotide (Sirajuddin et al. 2007; Zent et al. 2011; Macedo et al. 2013).

In addition to nucleotide binding, septin complexes show a slow GTP hydrolysis, however, rates of hydrolysis depend on the composition of the analyzed complex (Field et al. 1996; Farkasovsky et al. 2005; Huang et al. 2006). GTP-hydrolysis can be observed from yeast to human, proving it to be an important role in the function of septins. In 2014, Zent et al. have investigated the complex GTPase reaction of all members of the four human septin groups. They have observed similar hydrolysis reactions in three septin groups in the monomeric state while SEPT6 has no GTPase activity. SEPT7 forms a very tight G-interface dimer in the GDP-bound state. Additionally, the stability of the interface is dramatically decreased by replacing GDP with a nucleoside triphosphate, which is believed to have an influence in filament formation via SEPT7 (Zent and Wittinghofer 2014).

Interestingly, a number of studies report that septin oligomers contain one member from each subgroup and suggest subgroup-restricted binding preferences of mammalian septins (Nakahira et al. 2010; Sandrock et al. 2011). The group 7 seems to be unique, because SEPT7 is the only member of its subgroup, not only in humans but also in most other organisms. The absence of SEPT7 will lead to the loss of other septin proteins in homo- and heterooligomeric complexes, so it seems to be essential in the formation of filaments (Kinoshita et al. 2002). In hexamers, SEPT7 is in terminal position (Sirajuddin et al. 2007) and it is further associated with nonpolar linear septin filaments (Tada et al. 2007; Mostowy and Cossart 2012). Sellin et al. (2011) explored native assembly states and subunits in the human septin system. These data indicate that mammalian septins exist predominantly in the context of six or eight subunit heteromers that depends on SEPT7 for stability. According to the crystallographic studies the basic unit is SEPT 7:6:2:2:6:7 in a hexamer where SEPT2–SEPT2 and SEPT6-SEPT7 interactions occurred via N–C interfaces and SEPT2-SEPT6 and SEPT7–SEPT7 interactions occurred via the G–G interface. While SEPT2 and SEPT7 were associated with GDP in the hexamer, SEPT6 was GTP-bound, suggesting that proteins in the SEPT6 family are GTPase-deficient, as mentioned above (Sirajuddin et al. 2007).

The composition of the septin complex is cell-type specific and essential for certain functions. Kuo et al. (2015) have investigated that SEPT12 as well as SEPT9 can flank the SEPT2-6-7 hexamers to form octamers in mammalian sperm annulus (e.g., 12-7-6-2-2-6-7-12 or 12-7-6-4-4-6-7-12), suggesting a critical role in sperm motility. Interestingly, human SEPT9, due to variable mRNA splicing, exists as multiple isoforms that differ between tissues. Sellin et al. (2011) have shown that the SEPT9 expression level directs the hexamer-to-octamer ratio in myeloid K562 cells, and that the isoform composition and expression level together determine higher-order arrangements of septins.

Several human septins might be exchangeable in these complexes (Tada et al. 2007; Xie et al. 2007), the individual members within one septin subgroup can substitute for one of the others at the same position of the complex in vivo and in vitro (Nakahira et al. 2010; Sandrock et al. 2011). It has been suggested that SEPT2 can be replaced by SEPT5 (or SEPT1/SEPT4) and SEPT6 by SEPT11 (or possibly by SEPT8/SEPT10) in a SEPT2- SEPT6- SEPT7 complex (Kinoshita 2003a, b). Therefore, the existence of SEPT5/7/11 complexes do not contradict the existence of previously reported SEPT2/6/7 or SEPT7/9b/11 complexes, and is consistent with earlier findings that downregulation of SEPT7 decreased the expression of other members of the septin complex (Kinoshita et al. 2002; Kremer et al. 2005). The absence of SEPT7 leads to the loss of other septin proteins in homo- and heterooligomeric complexes, and this protein appears essential for the generation of filaments (Kinoshita et al. 2002), as mentioned above.

*Posttranslational modifications* SUMOylation (*s* mall *u* biquitin-related *mo* difier) that regulates the removal of old septin rings (Johnson and Blobel 1999), acetylation that likely has an important role in the ring to collar transition and in the localization of septin rings (Mitchell et al. 2011), and phosphorylation, the best characterized modification that might have a large influence on the morphogenesis and cytokinesis, have been reported in septin oligomers. There are posttranslational modification regions within the G domain, near the G interface, to modulate septin heteropolymer assembly and stability. The posttranslational modifications within the highly diverged N- and C-termini, might be the most important for the assembly of higher-order septin structures that are unique to individual organisms (Hernández-Rodríguez and Momamy 2012). According to Sandrock et al. (2011), mutations of a potential phosphorylation site within SEPT7 regulates the binding to all other septins. In cells deletion of SEPT7 or mutation of the interaction surface prevented SEPT9 association with the complex, suggesting that it interacts with SEPT7 (Kim et al. 2011; Sellin et al. 2012).

Higher order assembly of septin filaments are facilitated by the association of these oligomers to the cell membrane (Bridges et al. 2014). Based on the results, that SEPT5 and SEPT11 can colocalize and coimmunoprecipitate with SEPT7, and expression levels of both are decreased in SEPT7 deficient neurons (Tada et al. 2007; Xie et al. 2007), the existence of a SEPT5–SEPT7–SEPT11 complex in neuronal dendrites was suggested. This is consistent with an earlier finding that SEPT7 level is significantly decreased in homozygotic SEPT5 null mice (Peng et al. 2002).

Identification of *small molecules that inhibit septins* could help to explain the functions of septins under normal and pathological conditions. The plant growth regulator forchlorfenuron (FCF), a synthetic cytokinin is known to inhibit septin dynamics in vitro without affecting either actin or tubulin polymerization (Hu et al. 2008). The inhibition of septin organization by FCF and the related physiological effects have been investigated in several organisms. Septins of the human blood fluke *Schistosoma mansoni* and their interactions with forchlorfenuron was investigated by Zeraik et al. (2014). Application of FCF at 50–500 μM concentration led to rapid polymerization of filaments and paralysis under different culturing developmental stages of schistosomes, which was reversible upon removal of the cytokinin. The findings implicated a direct effect on muscle contraction due to septin stabilization that might be responsible for the reversible paralysis. In infected cultures with *Chlamydia trachomatis,* which is an obligate intracellular human pathogen that grows inside a membranous, cytosolic vacuole termed an inclusion, FCF administration reduced growth of the chlamydial inclusion substantially (Volceanov et al. 2014). However, off-target effects of FCF have also been published. In budding yeast cells FCF treatment inhibited growth and the proliferation but did not have detectable effect on septin structure or function. FCF potently inhibited ciliation and motility, and induced mitochondrial disorganization in *Tetrahymena thermophila* without apparent alterations in septin structure. Moreover, cultured mammalian cells treated with FCF displayed fragmented mitochondria (Heasley et al. 2014). The ability of FCF to modify cell proliferation and invasion was shown in breast cancer cells (Zhang et al. 2016). FCF dose-dependently depressed cell viability and colony formation in MCF7 and in a highly invasive breast cancer cell line MDA-MB-231, also induced apoptosis, while at 100 µM concentration FCF developed cytotoxicity. In silico analysis of FCF docking to all available crystal structures of septins indicates that FCF interacts preferentially with the nucleotide-binding pockets of septins (Angelis et al. 2014). The inhibitor is predicted to form hydrogen bonds with residues involved in GDP-binding and thus stabilizes septins by locking them into a conformation that mimics a nucleotide-bound state, preventing further binding and GTP hydrolysis. The effectiveness of this cytokin was also tested in human gastric epithelial (HGE-20) cancer cells, where septins and the receptor tyrosine kinase ErbB2 were highly expressed. Cell exposure to FCF significantly reduced colocalization of ErbB2 with septins, but increased the level of ubiquitylated ErbB2 at the plasma membrane. These data suggest that septins protect ErbB2 from ubiquitylation, endocytosis, and lysosomal degradation and provide a basis for the development of new ErbB2-targeting anti-cancer therapies (Markus et al. 2016).

## Cellular function of septin proteins

Besides the originally described diverse function of septins in cytokinesis (Hartwell 1971), growing number of observations confirmed their other biological functions. They were found to contribute to several cellular processes, to participate in the development and physiology of specific tissues and organs, and their potential role in various pathophysiological states has also been indicated.

They can have an impact—among others—on *neurotransmission* e.g. on glutamate uptake. In a primary culture of glial cells, the expression of a SEPT2 isoform was critical for the assembly of septin units, the isoform with a mutated domain reduced the level of glutamate uptake (Kinoshita et al. 2004). Septins are also able to control the localization of ion channels, their clustering, and the release of synaptic vesicles. SEPT3, 4, 5, 6, 7, and 11 have been suggested to affect the neurotransmitter release and uptake.

Interacting with other components of the cytoskeleton Septins also play a role in the support and regulation of *cell shape*, the rigidity of the cell and facilitate their migration (Tooley et al. 2009; Dolat et al. 2014a, b; Kremer et al. 2007; Kim et al. 2010; Gilden et al. 2012). In HeLa cells reduced rigidity was observed in the absence of SEPT2 or SEPT11 (Mostowy et al. 2011). In mouse T cells SEPT7 depletion results in significantly impaired morphology. Furthermore, septins can form *diffusion barriers* that control the localization of cellular proteins thus contributing to cellular compartmentalization.

Although septins neither present motor activity, nor force generation, due to their interactions with the mechanotransduction machinery, they are supposed to have a role in *mechanobiology* and in the regulation of mechanotransductional pathways (Calvo et al. 2015; Dolat et al. 2014a, b; Simi et al. 2018). Potentially affecting the conformation of stretch-sensitive ion channels, they might be able to modify the stretch-activated cellular responses and downstream mechanotransduction (Pardo-Pastor et al. 2018; Coste et al. 2010).

Because of their ability to modify the formation of actin filaments and membrane rigidity (Sirajuddin et al. 2007; Tanaka-Takiguchi et al. 2009), they are supposed to participate in the creation and maintenance of *curved cellular* configurations. These assumptions are confirmed by the localization of septins: they are highly expressed in cellular structures with high curvature that generate or are exposed to mechanical stress (the annulus of spermatozoa, the bases of cilia and dendrites, surrounding invasive bacteria; see e.g. Mostowy and Cossart (2012).

The first mammalian septin (SEPT1) was cloned from lymphocytes (Carol et al. 1990), even so the role of septins in the *immune system* was not intensively studied so far. The proper function of the immune system requires coordinated work of different specialized cell types. Septins, especially SEPT9 have been found to be essential for the development of T lymphocytes (Lassen et al. 2013). The membrane structure of T-cells and their migrating capacity were also shown to be affected by septins (SEPT6 and SEPT7) (Gilden et al. 2012).

The influence of septins on mammalian *embryogenesis* is not yet understood, but genetic deletion of SEPT7, SEPT9, and SEPT11 proved to be lethal in the embryonic phase, reflecting their essential role (Kinoshita 2008; Fuchtbauer et al. 2011; Roseler et al. 2011). During the early development of the *heart*, the first organ to form during embryogenesis, SEPT2, 6, 7, and 9 interacting with the elements of the cytokinetic machinery could regulate cardiac functions (Ahuja et al. 2006). In the vascular network, in *platelets* SEPT5 has been observed and identified as a negative regulator of the fusion of storage vesicules (α-granules) (Dent et al. 2002). In the DiGeorge syndrome, characterized by cardial impairment and lesions, SEPT5 is deleted (McKie et al. 1997). Bernard-Soulier syndrome, a rare autosomal recessive blood disorder is also connected to altered SEPT5 expression (Lopez et al. 1998). In primary mouse cardiac endothelial cells, Borg5 (Binder of the Rho GTPase 5, also known as CDC42EP1) association with septins in the perinuclear region and its colocalization with actomyosin fibres were shown. Both Borg5 deletion and SEPT7 knockdown resulted in a disruption of the perinuclear actomyosin and persistent directional migration thus affecting efficient microvascular angiogenesis (Liu et al. 2014). Several blood disorders are also connected to improper septin expression, e.g. acute myeloid leukemia, acute lymphoblastic leukemia, and mixed lineage leukemia. SEPT2, 4, 5, 6, 9, and 11 are potential key players of these diseases (Cerveira et al. 2011; Muntean and Hess 2012).

Septins are widely expressed in the *nervous system*. Their impact on neurotransmission was mentioned above. Their role in differentiation of neurites into axons and dendrites, neuronal migration, formation of dendritic spines, dendritic arborization, branching of axons were also revealed, and the involvement of SEPT4, 6, 7, 11, and 14 have been established (Shinoda et al. 2010; Tada et al. 2007; Xie et al. 2007; Cho et al. 2011; Hu et al. 2012). Since septins have essential roles in the development and activity of the nervous system, and they are also integral elements of neurotransmission, any impairment of their expression results in neurodegenerative disorders. Schizophrenia, bipolar disorders (SEPT5, 6, and 11) (Pennington et al. 2008), Down syndrome (SEPT4, 6, and 7) (Sitz et al. 2008), Alzheimer’s disease (SEPT1, 2, and 4) (Kinoshita et al. 1998), and Parkinson’s disease (SEPT4) (Ihara et al. 2007) were all associated to abnormalities of septin expression.

The function of the *reproductive system* has also been connected with septin expression. Septins were assigned to male infertility and ovarian cancer. SEPT4 (Ihara et al. 2005; Kissel et al. 2005), and in addition SEPT1, 6, 7, and 12 (SEPT12 is expressed only in the testis) account for the former (Toure et al. 2011; Lin et al. 2009), while SEPT9 has been found to contribute to the latter pathology (Kalikin et al. 2000; Russell et al. 2000).

The development and function of the *excretory system, gastrointestinal tract, and respiratory system* depend on the proper function of epithelial cells, which requires a well defined localisation of channels and transporter molecules in the membrane. SEPT2 has been proven to be necessary for the transport of vesicles from the Golgi apparatus to the membrane and for the structure of the cytoskeleton (Spiliotis et al. 2008; Bowen et al. 2011). Its deletion results in improper epithelial cell shape and membrane protein accumulation in the cytoplasm. SEPT2 and SEPT2/7/9 complexes regulate morphology of cilium that helps to monitor the flow of fluid in the nephron (Hu et al. 2010; Ghossoub et al. 2013). SEPT7 might influence the regulation of glucose transport in podocytes (Wasik et al. 2012). SEPT2 and 11 are overexpressed in renal cell carcinoma (Craven et al. 2006), SEPT4 in colorectal cancer (Zieger et al. 2000; Tanaka et al. 2001), while the level of SEPT9 decreases progressively during tumorigenesis due to gene methylation during colorectal cancer progression (Toth et al. 2011), consequently SEPT9 test is used to screen early-stage colon cancer (Warren et al. 2011). In the respiratory system, SEPT2 helps to sustain membrane integrity of shear-stressed epithelial cells (Sidhaye et al. 2011).

Septins are present in the *skin* as well. SEPT1, 4, 8, 11, and 14 were observed in epidermal keratinocytes. In squamous cell carcinoma and malignant melanoma upregulation of these septins and their interaction with the cytoskeletal elements were detected (Mizutani et al. 2013). However, the metastasis formation and malignant transformation are not yet unambiguously connected to septins.

## Interactions between septins and other cytoskeletal elements

Many recent studies have shown that septins fundamentally interact with actin-cytoskeleton, microtubules and membrane structures of cells, respectively. So, they might be considered as the 4th element of the cytoskeletal structure (Mostowy and Cossart 2012).

### Septin-microtubule interactions

The first result that led to the conclusion that septins may interact with microtubules was an inspection on *Drosophila*, where SEPT1 and SEPT2 were shown to be able to bind to microtubules under in vitro conditions (Sisson et al. 2000). Subsequent studies have shown that more than half of septin isoforms colocalize with microtubules and these associations exert potential role of septins in mitotic events (Silverman-Gavrila and Silverman-Gavrila 2008). A few septin variants like SEPT1, SEPT2, SEPT6, and SEPT9 are located at the mitotic spindle in the mitosis phase of the cell cycle, while these isoforms are found at midbody in the telophase of the mitosis (Qi et al. 2005; Spiliotis et al. 2005; Nagata et al. 2003). A further study confirmed the importance of the aforementioned septin variants in the mitotic events using gene silencing, where the lack of septins led to abnormalities in the cyto- and karyokinesis. Moreover, septins not only show co-localization with microtubules, but they are able to influence their dynamics through microtubule-associated protein 4 (MAP4) interaction, by linking with MAP4 and preventing its binding to the microtubules (Kremer et al. 2005). Furthermore, interactions between septins and microtubules have also been described in neuronal cells where microtubule-dependent cytoplasmic movements have crucial role in neurotransmission and axonal outgrowth (Neufeld and Rubin 1994). Meanwhile, SEPT3 is presumably involved in synaptic vesicule recycling, since it is primarily co-localized with synaptophysin and dynamine at the presynaptic terminal (Xue et al. 1989). On the other hand, SEPT2 is associated with tubulin and this interaction drives the vesicular transport in a MAP4-dependent manner (Ikegami et al. 2007).

### Septin–actin interactions

Experiments performed on *Drosophila* embryos have proven that septins are essential for normal cell shape formation as septins contribute to the evolvation of regular actin bundles (Mavrakis et al. 2014). Shortly after identification of mammalian septins, it was found that some septin isoforms as SEPT2, SEPT6, SEPT7, and SEPT9 show colocalization with the actin cytoskeleton (Joberty et al. 2001; Kinoshita et al. 1997; Surka et al. 2002; Xie et al. 1999). In non-dividing cells, the septins primarily interact with actin stress fibers (ASF) (Kremer et al. 2007; Dolat et al. 2014a, b) and their role is crucial in their spatial organization and function exemplified by the fact that depletion of septins results in shorter, less massive and stable ASFs, and these fibers are dispersed rapidly after their formation (Estey et al. 2010). Moreover, the absence of septins leads to mislayed stress fibers (SF) and reduced mechanotransduction and it is well characterized by the lack of focal adhesion, maturation, and reduced extracellular matrix (ECM) transformation and contraction (Kremer et al. 2007). In mitotic cells septins concentrate mainly at the cleavage furrow and take part in the development of the contractile and circular acto-myosin bundles during cell division (Estey et al. 2010; Schmidt and Nichols 2004). In adherent HeLa and MDCK (Madin-Darby Canine Kidney) cells SEPT2 depletion interferes with the process of cleavage furrows, which finally results in bi-nucleated cells (Schmidt and Nichols 2004). Recent studies propose that septins play a role in the preservation of the perinuclear actin matrix based on the presence of septin filaments on the ventral side of nuclei where they colocalize with actin (Verdier-Pinard et al. 2017). Although we lack detailed information about the localization of septins in branched actin mash, but it has already been proven that septins, especially SEPT1 and SEPT5 in carcinoma and melanoma cells are highly present in lamellopodia and are required for cell spreading (Mizutani et al. 2013). Furthermore, experiments performed on DRG (Dorsal Root Ganglion) neurons suggest robust expression of SEPT6 at branched actin that results in axonal filopodia (Hu et al. 2012).

### Septin–plasma membrane interactions

It has been shown that septins fundamentally interact with the lipid bilayer, especially with phospholipids, an association which is capable of influencing the organization of septins into filaments. Experiments performed in yeasts have confirmed that lower-order septin structures in are able to polymerize into filaments on PtdIns-4,5-bisphosphate-containing lipid monolayers (Bertin et al. 2012). In mammalian MDCK cells, septins have been shown to boost membrane fusion events that enhance the maturation of macropinosomes and promote lysosomal functions (Dolat and Spilitois 2016). Impressive results by Dolat and Spiliotis (2016) have demonstrated the original role of septins in endocytosis and the fusion of endomembranes. Septins localize the actin filaments to the plasma membrane, which affects the binding of actin to the membrane, or even modifies the linear or diversified polymerization of actin (Hagiwara et al. 2011). Although experimental data are not yet available, it is feasible that septins may be involved in the anchoring of mitochondria to membrane-derived intracellular organelles such as endoplasmic reticulum or Golgi, as it has been demonstrated that membrane proteins of these organelles interact with mitochondria directly or indirectly (Gurel et al. 2014).

## Septins and calcium signaling

The regulatory role of septins in intracellular calcium homeostasis was also investigated. Mutated *stim1* and *orai1* genes reduce SOCE in T-cells and generate severe combined immunodeficiency syndrome. Altered expression of these proteins is also involved in muscle differentiation defects and several human diseases like congenital progressive myopathy. There are evidences to show the regulatory function of septins in this process, however, septin subgroups can modulate SOCE differently in *Drosophila* flight circuit neurons (Deb and Hasan 2016). The SEPT2 subgroup (dSEPT1 and dSEPT4) functions as a positive regulator of SOCE, suggesting an evolutionary conserved role of the isoforms of this subgroup in different cell types. While reduction of dSEPT7 alongside with an IP_3_-receptor mutation increased extracellular Ca^2+^ uptake even without store depletion. In *Drosophila* neurons the interaction between SEPT7 and functional Orai channels was proved (Deb et al. 2016). According to the results from primarily cultured neurons the authors suggest that SEPT7 works as a molecular brake on the activation of Orai channels. The molecular reason suggested for these results is that dSEPT7 forms a complex with dSEPT1 and dSEPT2 which then maintains a break on dSTIM recruitment to the ER-PM regions.

In HeLa cells genome-wide RNA interference screen was used to identify new modulators of Ca^2+^/NFAT signalling (Sharma et al. 2013). NFAT (Nuclear factor of activated T-cells) is a transcription factor activated by sustained Ca^2+^ influx across plasma membrane, mainly through Orai channels. In septin depleted cells, where SEPT2, SEPT4, and SEPT5 were simultaneously targeted, physiological STIM1-mediated Orai activation was impaired, while Orai channel itself was intact and could be gated by soluble STIM1. The role of septins was also proved by the application of FCF which immobilizes septin filaments and produced inhibited Orai cluster formation, STIM1-Orai1 colocalization and impaired SOCE. They suggested that septins organize highly localized plasma membrane domains that are required for the efficient STIM1-Orai1 communication or other membrane microdomains relevant to different signaling processes.

It has been shown that members of the SEPT2 subgroup destabilize septin complexes in mammalian cells, while reduction of SEPT7 allows heterodimeric or heterotetrameric formation of SEPT2 and SEPT6 group members, but affect the formation of complexes via its terminal position within the assembly (Sellin et al. 2011). As SEPT2/4/5 subunits are necessary for nucleate septin complex formation, their reduction destabilizes septin filaments leading to abnormal Orai organization in resting cells and reduced STIM/Orai coupling and Orai opening after store depletion. However, reduced SEPT7 levels result only in shorter filaments and attenuate organization of membrane lipids into a permissive form for STIM/Orai coupling and Orai opening even in resting cells.

## Function of septin proteins in muscles

### Expression of septins in muscle in various organisms

Regarding the expression and role of septin proteins in muscle type tissues limited information is available. A novel cDNA that encodes a protein homologous to yeast cdc10 was isolated from human samples, and mapped to chromosome region 2q37 by fluorescent in situ hybridization. The peptide sequence contained a motif of the GTPase superfamily with conserved domains are rich in basic residues. Two different transcripts were shown, the major type, 3.5 kb long, was expressed ubiquitously in all human tissues examined, but a 2.0-kb alternative transcript lacking any long AU-rich element in the 3′ non-coding region was expressed abundantly only in testis, heart and skeletal muscle (Mori et al. 1996). In acute myeloid leukemia a gene was identified as a myeloid/lymphoid leukemia (MLL) fusion protein partner and was named MSF (MLL septin-like fusion). Two human alternative transcript (MSF-A and MSF-B), and a third database variant of MSF (MSF-C) were identified from adult and fetal tissues; a 4.0-kb transcript MSF-B was detected only in skeletal muscle, the appropiate protein of 422 amino acids included a conserved GTPase domain and a xylose isomerase 1 domain, indicating that MSFs are involved in the complex metabolic regulation in different tissues (Kalikin et al. 2000).

Conserved SEPT7 expression was proved in both embryonic and adult zebrafish hearts reflecting to its role in the developmental immaturity of the zebrafish heart compared with mammals. In adult hearts SEPT7 expression could mainly be detected in the endothelium and not directly in the cardiomyocytes (Gomes et al. 2016). The SEPT7 (cdc10) gene has been shown to be expressed at different levels in the longissimus muscle (LM) between low-marbled and high-marbled steer groups. It is located within the genomic region of a quantitative trait locus for marbling, and its expression level was shown to be positively correlated in LM with marbling in Japanese Black (JB) steers (Tong et al. 2015b). High expression levels of SEPT2, SEPT6, SEPT7, and SEPT9 were shown in mice embryonic heart, while their level significantly decreased around birth and was not detectable in adult heart tissue (Ahuja et al. 2006). High-throughput analysis of the potential genes related to blood pressure regulation was conducted from aorta, liver, heart, and kidney samples of a mouse model. As a result, sept6 and pigx were found in the top 10 high blood pressure (BPH)- related differentially regulated genes (DEGs), indicating their roles in the pathogenesis of this common chronic disease (Zhu et al. 2018).

### Functional studies of septins linked to muscle

Knockdown of SEPT7 significantly reduced F-actin and α-cardiac actin expression and thus caused myofibrillar disorganization and disruption of sarcomere structure in zebrafish heart. Depletion of SEPT7 decreased the expression of retinaldehyde dehydrogenase 2, which is essential for the retinoic acid synthesis and thus necessary for the morphogenesis of the heart. Functionally, the aforementioned genetic alteration reduced the ventricular dimensions, contractility and cardiac output without changing intracellular Ca^2+^ tranzients (Dash et al. 2017). Also in zebrafish, SEPT7 knockdown altered myosin heavy chain localization and disorganization of muscle fibers in somitic muscle presumably due to depletion of SEPT7 from myotendinous junction, the contact sites between muscles and tendons (Dash et al. 2017).

Functional interactions of SEPT2 were also investigated using specific Rho kinase or actin and myosin inhibitors suggesting a strong correlation of septin expression and the ability of cardiomyocytes to undergo cytokinesis (Ahuja et al. 2006). A study, where cardiovascular toxicity of non-steroidal anti-inflammatory drugs were tested in cultured cardiomyocytes revealed that many proteins involved in membrane organization were affected. Among them, SEPT8, a filament scaffolding protein, showed lower expression levels in cells treated with drugs of relatively high toxicity such as celecoxib, diclofenac, valdecoxib, and rofecoxib. In contrast, SEPT8 expression was unaffected in the presence of less toxic drugs as ibuprofen, naproxen, and meloxicam (Baek et al. 2010). In cultured C2C12 cells, a model system of skeletal muscle, downregulation of SEPT7 expression resulted in significant changes in cell morphology, decreased cell proliferation, and markedly reduced differentiation process of the cells (Angyal et al. 2019). In vivo, skeletal muscle-specific, inducible reduction of SEPT7 protein expression in mice severly modified muscle architecture. The individual myofibrils became smaller but their number increased in SEPT7 KD mice. However, the reduced SEPT7 expression was not associated with altered excitation–contraction coupling machinery, but significantly modified tissue repair in BaCl_2_-injected *Tibialis posterior* muscle (Dobrosi et al. 2019). These findings suggest that special septin isoforms present in striated muscles could have an essential role in determining normal physiological properties, differentiation, and muscle regeneration.

Furthermore, not only the expression pattern of different septin isoforms, but genetic variations (e.g. small nucleotide polymorphism, SNPs) within the appropriate genes can influence the physiological role of septins. SNP in the promoter region of the cdc10 gene was shown to affect marbling in JB cattle (Tong et al. 2015a, b). A high number of SNPs are already known in septin genes, however their direct or indirect correlation with any disease has not to our knowledge been studied so far. Most of the possible SNPs in mouse SEPT7 gene are localized in the coiled-coil region, and none in the polybasic region or within the G1, G3, and G4 motifs.

## Conclusion

Striated muscles are perfectly designed machines responsible for contration (Henderson et al. 2018). The complex cytoskeletal networks are critical for the coordination of the contractile activity of the sarcomere. This basic contractile unit is comprised of precisely organized individual filament system including thin (actin), thick (myosin), titin, and nebulin. Intermediate filaments serve as a connection between the sarcomeres and other organelles (mitochondria and nucleus) and are responsible for the cellular integrity. The connection site of sarcomeres and cell membrane (also called costamer) participates in the synchronization and transmission of muscle force. Proteins involved in these cytoskeletal assemblies are intensively investigated, mutations within their genes contribute to a number of human myopathies, however, the detailed functions and interconnections still have to be cleared. One of the major missing parts is to clarify the possible role of septins in striated muscle tissues. Emerging evidence suggest an important function for septins as a cytoskeletal protein in cardiac and skeletal muscle, similarly to their contribution in different cellular processes in non-exitable cell types. Recently published data revealed that SEPT7 has an essential role in cultured muscle cells during differentiation in vitro and in determining skeletal muscle architecture in vivo. Despite the myriad of information collected on the tissue specific function of septins, their precise role in the pathology of the human diseases needs further investigation. They are already used as biomarkers, but their use as a therapeutic target requires additional research.
